# The effects of dual task training to improve self-efficacy, functional performance, and quality of life in individuals with Parkinson’s disease: A study protocol

**DOI:** 10.1371/journal.pone.0362454

**Published:** 2026-11-12

**Authors:** Alvaro Reyes-Ponce, Travis Cruickshank, Andrea Mira, Nuria Pérez-Romero, Josefa Domingos

**Affiliations:** 1 Exercise and Rehabilitation Sciences Laboratory, School of Physical Therapy, Faculty of Rehabilitation Sciences, Universidad Andres Bello, Santiago, Chile; 2 Research and Innovation Office, Murdoch University, Murdoch, Perth, Australia; 3 Centre for Precision Health, Edith Cowan University, Perth, Australia; 4 Exercise and Rehabilitation Sciences Institute, School of Occupational Therapy, Faculty of Rehabilitation Sciences, Universidad Andres Bello, Santiago, Chile; 5 Exercise and Rehabilitation Sciences Institute, Postgraduate, Faculty of Rehabilitation Sciences, Universidad Andres Bello, Santiago, Chile; 6 Egas Moniz Center for Interdisciplinary Research (CiiEM), Egas Moniz School of Health & Science, Almada, Portugal; PLOS: Public Library of Science, UNITED STATES OF AMERICA

## Abstract

**Introduction:**

Dual tasking is impaired in Parkinson’s disease (PD) and adversely impacts activities of daily living and quality of life. The benefits of different dual-task training modalities and the prioritization strategy (directing attention to both tasks versus shifting between them) in individuals with or without cognitive impairment are unclear. It is also unknown whether motor-cognitive benefits from dual-task training are associated with reduced attentional demand. In addition, lower perceived ability to succeed at a specific task (self-efficacy) is associated with worse motor and non-motor symptoms and quality of life. Higher dual-task performance may be associated with higher self-efficacy, which in turn improves dual-task performance. This protocol describes a study evaluating the effectiveness of different dual-task training modalities (motor-motor vs. motor-cognitive) and attentional prioritization strategies (fixed vs. variable) on self-efficacy, functional performance, and quality of life in people with PD, with and without cognitive impairments.

**Methodology:**

Using a factorial design nested within cognitive status, participants will be randomized into one of four groups combining task type (motor-cognitive or motor-motor) with priority instruction (fixed or variable). An interdisciplinary team will design individualized programs. In fixed-priority training, attention is maintained on both tasks, in variable-priority training, attention alternates, focusing on the motor task for half the session and on the secondary task for the remainder. All participants will complete 24 individual sessions (3x/week for 8 weeks, 60 minutes each). Outcomes include self-efficacy, motor and cognitive performance, quality of life, and prefrontal cortex activity.

**Expected results:**

This protocol will introduce a person-centered approach to dual-task training, comparing modalities and attentional strategies tailored to cognitive status. It also addresses a key gap by examining neurophysiological mechanisms of dual-task interference through real-time brain activity, potentially identifying biomarkers linking neural changes to clinical outcomes. Findings will inform targeted, evidence-based neurorehabilitation strategies for PD.

**Protocol registration:**

The protocol of this study is registered in Clinical Trials Registry with a registration number NCT07611578. Date registered: 05/07/2026. https://clinicaltrials.gov/study/NCT07611578?id=NCT07611578&viewType=Card&rank=1

## Introduction

Parkinson’s disease (PD) is a chronic and progressive neurodegenerative disorder associated with the early death of a specific group of neurons in the brain that regulates the neurotransmitter dopamine [1–3]. The degeneration of dopaminergic cells in individuals with PD leads to a progressive decline in their ability to control automatic movements involved in day-to-day activities (e.g., waking, talking, picking up a cup to drink) [4,5]. Consequently, individuals with PD, when performing two tasks simultaneously (one motor and one cognitive or two motor tasks), have fewer attentional resources to perform the secondary task [6]. Attention is a limited resource, and dividing attention between two tasks performed concurrently can negatively impact performance in one or both tasks [7]. This phenomenon, known as dual-task interference or dual-task effect, refers to the change in performance when performing two tasks (dual tasking) compared to performing a single task (single tasking) [8,9]. Under dual tasking, studies reveal that individuals with PD exhibit reduced balance control [9,10], decreased gait speed, stride length, and cadence [11–13], reduced cognitive performance (executive function, spatial memory, calculation and reaction time) [14–16], reduced speed and amplitude during handwriting [17] and reduced mobility [18]. Furthermore, individuals with PD and mild cognitive impairments demonstrate greater dual-task interference than those without cognitive impairments [2]. Individuals with PD may then avoid dividing attention between simultaneous tasks during daily activities for fear of falling or embarrassment in a cognitive task. Precisely, higher fear of falling has been revealed to be associated with higher cognitive impairments [19]. Individuals with PD experience restricted social participation, autonomy and self-efficacy in relation to healthy older adults that contribute to poor quality of life [12]. Self-efficacy is the perceived ability to perform specific tasks to achieve concrete goals successfully [13]. Specifically in individuals with PD, lower self-efficacy is associated with worse motor and non-motor symptoms and lower quality of life [15] Self-efficacy and rehabilitation activities are reciprocally connected, so that an individual with high self-efficacy in performing a task will exhibit higher performance in the task, which in turn will increase self-efficacy and motivation with the rehabilitation [17].

In individuals with PD, dual task cost or interference in performing two tasks is higher during motor-cognitive dual tasking than in motor dual tasking. This suggests that motor-cognitive dual tasking may be more challenging [20]. Which dual task-training modality is more beneficial in individuals with PD with and without cognitive impairments is unknown. Whether or how a secondary cognitive or motor task produces more interference on a primary motor task depending on the cognitive status of individuals with PD has not been studied. It is unclear if greater interference of a secondary task is associated with greater benefits after dual task- training.

During dual task training, individuals can be instructed to direct attention to both tasks (fixed priority instructions) or to shift attention between tasks (variable priority instructions). Evidence indicates that motor performance deteriorates when individuals with PD are instructed to pay equal attention to both tasks, and it improves when they are instructed to prioritize the primary over the secondary task [21]. Furthermore, the instruction of attentional allocation may have different effects on individuals with PD depending on their cognitive level. Research suggests that individuals with PD and mild cognitive impairment tend to prioritize cognitive tasks over posture, whereas those with PD and no cognitive impairment prioritize posture over cognitive tasks [16]. This prioritization strategy may increase the risk of falls in PD patients with cognitive impairments, particularly in cases when prioritization instructions are not explicit. The more beneficial strategy for individuals with PD and cognitive impairments also remains unclear.

Given that individuals with PD have reduced capacity to perform automatic movements, they are forced to use extra attentional resources of the prefrontal cortex to perform dual task activities safely. Therefore, in individuals with PD, higher neuronal activity in the prefrontal cortex during dual tasking indicates increased oxygen metabolism and demand, over-recruitment of this brain area, and reduced efficiency in executing dual tasks [22]. Functional NIRS has been shown to be feasible, well tolerated and sensitive technique to measure neuronal activity of the prefrontal cortex during dual tasking in individuals with PD [23]. Examining neuronal activity of the prefrontal cortex in individuals with and without cognitive impairments under single and dual task conditions will provide a better understanding of physiological changes in the brains of individuals with PD.

This study compares different dual-task training modalities in individuals with PD. Specifically this study tests whether motor-motor or motor-cognitive dual-task training and fixed or variable priority instructions produce greater improvements in functional performance, quality of life and self-efficacy. We also examine whether cognitive status determines which modality is most effective. Finally, our purpose is to determine whether improvements in functional performance, quality of life and self-efficacy are associated with changes in motor and cognitive function, cortical activity and patient reported outcomes.

## Methods

### Experimental design

This study protocol will employ a (2x2) factorial experimental design nested in a factor variable (cognitive impairment). A description for the schedule of enrolment, interventions and assessments is presented in Fig 1. The study will follow the guidelines of the SPIRIT statement [24]. Participants with PD will be allocated to each group using the block randomization method. Allocation concealment will be implemented using sequential sealed envelopes prepared by a research assistant independent of the research team. The intervention will be delivered individually to each participant by at least two trained therapists with experience in dual task training. The therapists will be specifically dedicated to providing the interventions. Therapists will provide dual task training following planning by the interdisciplinary group, informed by baseline assessments**.** A research assistant will notify the therapists by emailing the group allocation to ensure concealment. Data will be collected by researchers blinded to group allocation. All participants will be assessed at baseline and after 24 sessions of dual task training.

**Fig 1:**
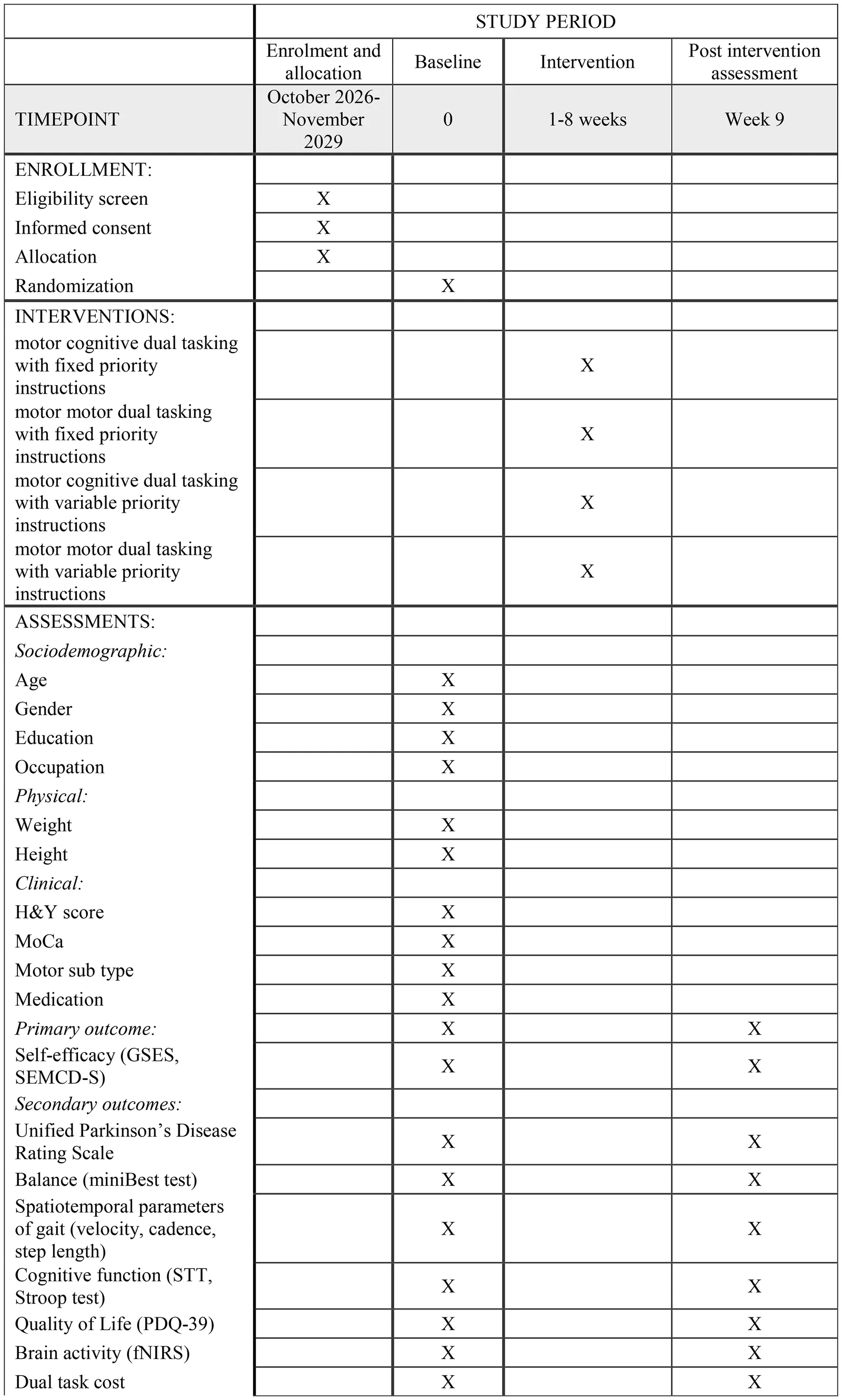
SPIRIT diagram for the timeline of enrolment, interventions, and assessments. Abbreviations: MoCa: Montreal cognitive assessment, GSES: General Self-Efficacy scale, SEMCD-S: Self-Efficacy to Manage Chronic Disease scale, STT: serial threes test, PDQ-39: Spanish version of the Parkinson’s disease questionnaire, fNIRS: functional Near-Infrared Spectroscopy.

### Participants

Participants diagnosed with idiopathic Parkinson’s disease confirmed by a neurologist will be recruited for this study [25]. Inclusion criteria will be: 1) Hoehn & Yahr stages I-III, 2), participants with no or mild to moderate neurocognitive impairments, 3) participants with and with-out symptoms of depression, 4) able to walk independently, 5) unaltered dose and type of medication up to three months before the commencement of the study, 6) participants able to understand and respond to the instructions given in the study. Exclusion criteria will be: 1) the presence of any other concomitant neurological or musculoskeletal disease, 2) history of leg or brain trauma or surgery, 3) vision or hearing problems that may affect the performance of cognitive tests, and 4) clinical diagnosis of major neurocognitive disorder. Neurocognitive disorder will be determined by the criterion defined by *The Diagnostic and Statistical Manual of Mental Disorders, Fifth Edition, Text Revision (DSM-5 -TR)* previously diagnosed by a specialist. The 15-item Geriatric Depression Scale will be used for depression symptoms screening because it has shown the highest diagnostic sensitivity [26]. Motor subtype of participants (e.g., tremor-dominant, postural instability gait disorder, freezing of gait, etcetera) will be recorded for sub-group analysis. All medications relevant to the disease (e.g., anticholinergics, benzodiazepines) will be also recorded as they have the potential to interfere with executive functions. The study was approved by the Human Research Ethics Committee at Andres Bello University (#032/2006). All participants will be given a written information document, and informed consent will be obtained before the commencement of the study.

### Sample size calculation

The sample size calculation was informed by Strouwen et al., 2017 and Valenzuela et. al., 2020 [27,28] which reported dual-task cost of motor and cognitive performance during concurrent motor-cognitive tasks. Based on the results of these studies we anticipate a difference of at least 12% between dual tasking training modality and assume a large effect size (Cohen’s f = 0,40) for post intervention gait velocity under dual-task conditions. Statistical power was set at 0.8 and the alpha level at 0.05 with two tails. For a 2x2 factorial experimental design nested in the cognitive status of participants, to examine the effects of dual-task training modality, priority instructions, time (pre post intervention) and interactions it was estimated that a sample size of at least 12 participants per group (8 groups) and 96 in total. We also estimate a drop-out rate of 13% per group. Therefore, we aim to recruit 14 participants per group and 112 in total over a period of 2.5 years.

### Procedures

Participants will be recruited from October 2026 to November 2029. Data collection will be completed in January 2030, and results are expected by March 2030. The purpose and nature of the study will be explained to all individuals interested in the study. Written informed consent will be obtained from all participants who fulfilled the eligibility criteria. Before the commencement of the intervention, all participants will be interviewed regarding their general health status. Sociodemographic data and information regarding each participant’s PD will be recorded. All outcomes will be measured before and after 24 sessions of dual task training by researchers blinded to group allocation. Baseline measurement will be performed by the interdisciplinary group of physical therapist, occupational therapist and psychologist. Participants will be familiarized with the assessments before data collection. After familiarization, cognitive and motor functions under single and dual-task conditions will be repeated three times to assess the test-retest reliability. Before data collection, the interdisciplinary team will perform rehearsal testing sessions for all the assessments to standardize the procedures, criteria, and results’ interpretation and ensure data quality. Assessments will be conducted during the “on” state, at a fixed interval of 60–90 minutes after the participant`s usual levodopa dose, consistent across pre and post intervention assessment for each participant. Participants will be instructed to take their medication at their usual time on assessment days, and time since last dose will be recorded. For all participants, levodopa equivalent daily dose (LEDD) will be calculated using standard conversion formulas, and concomitant medication classes will be recorded (MAO-B inhibitors, dopamine agonists, amantadine, and others), given their differential effects on gait, cognition, and impulsivity. LEDD and medication class will be reported descriptively by group and considered as covariates. At the start of each 60-minute session, participants’ motor state will be screened, and clinically significant wearing-off or dyskinesia occurring mid-session will be documented using the MDS-UPDRS Part IV. If wearing-off compromises safety (e.g., increased fall risk) or ability to perform the task, the session will be paused, and difficulty level will be reduced or the session shortened; the actual training time in a functional “on” state will be recorded per session as a measure of effective training dose. Sessions substantially affected by motor fluctuations will be flagged and considered in the analyses.

Before the commencement of the intervention, an occupational therapist will administer the Canadian Occupational Performance Measure (COPM). During the interview, participants will select three to five activities they find challenging and wish to improve. Participants will rate these activities on a scale from 1 to 10, evaluating both their perceived performance ability and their satisfaction with it. In individuals with PD the COPM has demonstrated to be useful, valid and reliable [29,30]. This instrument has an official and validated version in Spanish. In addition to obtaining baseline COPM scores, the information gathered during the interview will be used by the interdisciplinary team to identify patient-centred rehabilitation goals and to individualize the dual-task training program.

### Intervention (dual task training)

All participants will receive 24 sessions of dual task training, 3 sessions per week, for 8 weeks for 60 minutes each session. Dual task training will be delivered individually to each participant. Therapists will deliver dual-task training based on the assessments and program individually designed by the interdisciplinary team at baseline. Participants in the motor-cognitive task training will be instructed to perform motor tasks while simultaneously performing cognitive tasks. Examples of motor cognitive tasks are walking or balancing while spelling words forward and backwards, walking/balancing while naming objects starting with a specific letter, walking/balancing, and summing and counting or walking/balancing while doing go no go tasks. Cognitive tasks will be drawn from a predefined battery spanning several domains, such as attention, working memory, inhibition, flexibility, perceptual performance, mental tracking, reaction time and executive performance. Motor tasks include walking forward or backwards, walking in tandem and walking on an S-shaped route, transfers from sitting to standing, rolling from supine to sitting and from sitting to supine. Participants in the motor dual task training will be instructed to perform two motor tasks simultaneously. Examples of dual motor tasks are walking while holding a tray with or without glasses, walking while bouncing balls of different diameters, walking while throwing and catching a ball. Motor-cognitive training will include activities of daily living and functional performance of occupations that are meaningful and relevant for each person. Therapists will calibrate each participant’s starting task and difficulty level at baseline to their cognitive profile and educational background. Participants under variable priority instructions will be trained to focus attention on walking performance during half of the training session and in the rest of the session to focus on the performance of the secondary cognitive or motor task depending on group allocation. Participants under fixed priority instructions will be trained to maintain attention on both tasks (motor and cognitive or motor and motor) through the training session. Gait variability will be recorded as a proxy to verify that instructions for fixed and variable priority instructions are met. Verbal instructions for the prioritization (fixed versus variable) will be scripted verbatim. Cueing frequency will be capped and standardized across groups (fixed reminder cues, identical in number and timing across conditions; only cue content will differ between fixed and variable priority instructions). In all groups, dual task exercises will be standardized as well as the progression and the difficulty of both tasks. To design dual task training, motor and cognitive tasks will be selected and adapted based on baseline measurements, functional necessities of each participant progression in the exercise, and patient centered goals. The training protocol will include bidirectional difficulty adjustment. That is when performance criteria are met (at least 80% of success in motor and cognitive tasks performed in two consecutive session) but reduced or maintain difficulty when performance drops (less than 50% of motor and cognitive tasks performed successfully in two consecutive sessions), or when participants report factors such as poor sleep, medication changes, or a symptomatic ‘off’ day. Every adjustment, along with its direction and rationale will be logged at each session. Participants will be asked to rate the perceived level of fatigue and enjoyment (in a scale from 0 to 10) at the end of each training session to analyze potential differences in the level of complexity of each study arm.

### Measurements

#### Self-efficacy.

The Latin Spanish version of the Self-Efficacy to Manage Chronic Disease for the Chilean population (SEMCD-S) [31] and the General Self-Efficacy scale (GSES) [32] will be used for this outcome. In the SEMCD-S participants rank each one of the 4 items from 0 to 10. Higher scores indicate higher self-efficacy. In the GSES participants rank each one of the 10 items from 0 to 7. Similarly, higher scores indicate higher self-efficacy. Both instruments have revealed to be useful, valid and reliable with validated versions in Spanish.

#### Unified Parkinson’s Disease Rating Scale.

Part II and III of the Unified Parkinson’s Disease Rating Scale will be measured to analyse the effect of the intervention on activities of daily life and clinical motor aspects of the disease.

#### Balance.

The Mini BesTest will be used to assess static and dynamic balance during functional activities. The Mini BesTest has demonstrated to be valid and reliable clinical measure for assessing balance in individuals with Parkinson’s disease. The Mini BesTest has also been validated in Chile [33].

#### Gait.

Spatiotemporal gait parameters under single and dual-task conditions will be measured using a wearable inertial sensor (G-WALK, BTS Bioengineering Italy). During the single-task condition, participants will be asked to walk at a self-selected walking speed covering 10m. Under dual-task condition, participants will be asked to perform the same as above while performing cognitive tasks. Walking speed (m/s), cadence (steps/min) and stride length (cm) will be recorded for analysis.

#### Cognitive functions.

The Serial Threes Test (STT) and the visual Stroop task will be used to evaluate cognitive functions under single and dual-task conditions. The STT requires participants to make multiple subtractions of three from a whole three-digit number, *e.g.*, 256, 253, 250, 247. Different three-digit numbers will be used for each trial to reduce the learning effect. The visual Stroop task will require participants to respond (click on a computer mouse) to congruent and incongruent stimuli presented on a screen. The words “left” or “right” will be displayed on a TV screen, and participant will be asked to ignore the spatial position on the screen and instead indicate its semantic content by clicking the corresponding button.

#### Quality of life.

Quality of life will be measured using the Spanish version of the Parkinson´s disease questionnaire (PDQ-39). This instrument includes 39 questions distributed in eight domains where 0% is no problem and 100% is maximum level of problem [34]. This instrument has an official version for Chilean population.

#### Brain activity of the Prefrontal cortex.

The hemodynamic activity of the dorso and ventrolateral prefrontal cortex will be measured using a functional Near-Infrared Spectroscopy (Portalite, Artinis Medical Systems, Elst, the Netherlands) system under single and dual-task conditions. Two devices will be placed on the forehead of each participant, on the right and left side. In the sagittal plane, both detectors will be placed at 10% of the length of the nasion-inion starting from the nasion. In the transverse plane each detector will be placed at 7% of the head circumference starting from the midline. The devices will be placed to target left and right Brodmann`s areas 9 and 10. The devices will be protected against ambient light using a black headband covering the entire forehead. Raw data will be converted to relative changes in oxygenated and deoxygenated haemoglobin concentrations using the modified Lambert-Beer law.

#### Dual task cost.

Motor and cognitive outcomes will be measured under single and dual-task conditions. Under single cognitive task individuals will be asked to perform two tasks: the serial threes test (STT) and the visual Stroop task. The number of correct responses will be recorded for analysis in both tasks. For single motor task, individuals with PD will be instructed to walk at a self-selected speed covering 10m. Walking speed (m/s) will be recorded for analysis. Under dual tasking, the motor task will be combined with a secondary cognitive task (serial threes test and the visual Stroop task) and a secondary motor task (different from the motor tasks used for training) to be executed simultaneously. Single and dual tasks will be performed randomly to prevent the learning effect. Single and dual tasks will be repeated three times to assess test-retest reliability. The average of three trails will be calculated for analysis. Brain activity of the prefrontal cortex will be measured using the fNIRS system under single and dual-task conditions. Dual-task cost values will be calculated to compare the performance on dual task modality using the following formula:


$$\begin{array}{c} dual\;task\;cost(\%)=\frac{dual\;task\;performance-single\;task\;performance}{single\;task\;performance}x\text{1}\text{0}\text{0} \end{array}$$

A negative value indicate that the performance deteriorates in dual task condition relative to single task condition. A positive value indicates a relative improvement in dual task in relation to the single task condition.

### Statistical analysis

Descriptive data will be given as mean and standard deviation. A one-way analysis of variance and a Scheffé post hoc test will be performed to compare physical and physiological characteristics and baseline measurements between groups. To assess the test-retest reliability of motor and cognitive tasks under single and dual-task conditions, the interclass correlation coefficient and the coefficient of variation will be calculated for each group. Normality assumption will be tested using the Shapiro-Wilk test. Changes in outcomes measures after the intervention will be tested using a linear mixed model. The model will include self-efficacy, motor and cognitive function outcomes, quality of life, brain activity of the prefrontal cortex as response variable. Time (pre versus post intervention), group and interaction (group x time) will be fitted as explanatory variables. Group will be a fixed effect factor and time a random effect factor. Changes in patient reported outcomes will be analyzed using the Friedman’s and Wilcoxon rank-sum tests. Correlation between the amount of interference (dual task cost), patient reported outcomes and brain activity will be assessed using the Pearson and Spearman correlation coefficients. Statistical significance will be set at *P* ≤ 0.05. Statistical analyses will be performed using STATA statistical software version 18.0 and R version 4.3.2.

## Discussion

Parkinson’s disease is the fastest growing neurological disorder in prevalence, disability and death worldwide [35]. Chile is the Latin American country with the highest rise in PD prevalence and aging is considered a significant contributor to the increasing prevalence of the disease [36]. To date there is no cure for PD and therefore there is a growing demand in the PD community for accessing person centered therapeutic interventions that have an impact in improving quality of life.

Parkinson’s disease entails progressive loss of autonomy, often leading to learned helplessness, anxiety, and depression. Despite optimal medication management, motor and non-motor symptoms progressively impair functional independence and self-efficacy. Diminished self-efficacy is associated with reduced engagement in physical activity, avoidance of complex or novel tasks, and heightened fear of falling; factors that exacerbate physical decline and social isolation. Targeting self-efficacy within rehabilitation programs enhances participants’ perceived control over their condition, improves coping mechanisms, and promotes resilience. Therefore, self-efficacy becomes a critical determinant of long-term psychological adjustment and quality of life. However, there are no proven strategies to improve self-efficacy in individuals with PD. Dual tasking interventions have the potential to improve motor and cognitive function with a positive impact in improving self-efficacy. With the proposed research, our goal is to provide person centered, enjoyable and tailored dual task training that has a significant clinical impact on self-efficacy and quality of life in individuals with PD.

The neurophysiological mechanisms underlying dual-task interference in individuals with PD remain insufficiently characterized. While dual-task training modalities are used to improve motor cognitive function in individuals PD, the specific patterns of brain activity associated with distinct dual-task modalities are unknown. This knowledge gap is particularly pronounced in individuals with PD and cognitive impairments, in whom dual-task performance is markedly reduced. Critically, it remains unknown whether alterations in brain activity during dual tasking are associated with clinically meaningful outcomes such as self-efficacy. Advancing the understanding of the neural correlates of dual-task costs is essential to uncover the underlying neurophysiological substrates of cognitive-motor interference in PD. Such insights could inform the development of targeted neurorehabilitation strategies and serve as potential biomarkers for disease progression and intervention efficacy.

## Supporting information

S1 File SPIRIT 2025 checklist: Recommended items to address in a clinical trial protocol. (DOCX)

S2 File Original study protocol approved by the ethics committee. (PDF)

## Abbreviations

COPM: Canadian Occupational Performance Measure
DSM-5 -TR: Diagnostic and Statistical Manual of Mental Disorders, Fifth Edition, Text Revision
fNIRS: functional Near-Infrared Spectroscopy
GSES: General Self-Efficacy scale
LEDD: Levodopa equivalent daily dose
PD: Parkinson’s disease
PDQ-39: Spanish version of the Parkinson´s disease questionnaire
STT: Serial Threes Test
SEMCD-S: Self-Efficacy to Manage Chronic Disease scale.

## References

[1] Radder DLM, Lígia Silva de Lima A, Domingos J, Keus SHJ, van Nimwegen M, Bloem BR, et al. Physiotherapy in Parkinson’s disease: A meta-analysis of present treatment modalities. Neurorehabil Neural Repair. 2020;34(10):871–80. doi: 10.1177/1545968320952799 32917125 PMC7564288

[2] Kalia LV, Lang AE. Parkinson’s disease. Lancet. 2015;386(9996):896–912. doi: 10.1016/S0140-6736(14)61393-3 25904081

[3] Vijiaratnam N, Simuni T, Bandmann O, Morris HR, Foltynie T. Progress towards therapies for disease modification in Parkinson’s disease. Lancet Neurol. 2021;20(7):559–72. doi: 10.1016/S1474-4422(21)00061-2 34146514

[4] Brauer SG, Morris ME. Can people with Parkinson’s disease improve dual tasking when walking?. Gait Posture. 2010;31(2):229–33. doi: 10.1016/j.gaitpost.2009.10.011 19969461

[5] Intzandt B, Beck EN, Silveira CRA. The effects of exercise on cognition and gait in Parkinson’s disease: A scoping review. Neurosci Biobehav Rev. 2018;95:136–69. doi: 10.1016/j.neubiorev.2018.09.018 30291852

[6] Brauer SG, Woollacott MH, Lamont R, Clewett S, O’Sullivan J, Silburn P, et al. Single and dual task gait training in people with Parkinson’s disease: A protocol for a randomised controlled trial. BMC Neurol. 2011;11:90. doi: 10.1186/1471-2377-11-90 21791117 PMC3155494

[7] Dirnberger G, Jahanshahi M. Executive dysfunction in Parkinson’s disease: a review. J Neuropsychol. 2013;7(2):193–224. doi: 10.1111/jnp.12028 24007368

[8] Leone C, Feys P, Moumdjian L, D’Amico E, Zappia M, Patti F. Cognitive-motor dual-task interference: A systematic review of neural correlates. Neurosci Biobehav Rev. 2017;75:348–60. doi: 10.1016/j.neubiorev.2017.01.010 28104413

[9] Fernandes Â, Coelho T, Vitória A, Ferreira A, Santos R, Rocha N, et al. Standing balance in individuals with Parkinson’s disease during single and dual-task conditions. Gait Posture. 2015;42(3):323–8. doi: 10.1016/j.gaitpost.2015.06.188 26149283

[10] Bloem BR, Valkenburg VV, Slabbekoorn M, Van Dijk JG. The multiple tasks test. Strategies in Parkinson’s disease. Exp Brain Res. 2001;137(3–4):478–86. doi: 10.1007/s002210000672 11355392

[11] Bond JM, Morris M. Goal-directed secondary motor tasks: Their effects on gait in subjects with Parkinson disease. Arch Phys Med Rehabil. 2000;81(1):110–6. doi: 10.1016/s0003-9993(00)90230-2 10638885

[12] O’Shea S, Morris ME, Iansek R. Dual task interference during gait in people with Parkinson disease: Effects of motor versus cognitive secondary tasks. Phys Ther. 2002;82(9):888–97. doi: 10.1093/ptj/82.9.888 12201803

[13] Rochester L, Hetherington V, Jones D, Nieuwboer A, Willems A-M, Kwakkel G, et al. Attending to the task: Interference effects of functional tasks on walking in Parkinson’s disease and the roles of cognition, depression, fatigue, and balance. Arch Phys Med Rehabil. 2004;85(10):1578–85. doi: 10.1016/j.apmr.2004.01.025 15468014

[14] Salazar RD, Ren X, Ellis TD, Toraif N, Barthelemy OJ, Neargarder S, et al. Dual tasking in Parkinson’s disease: Cognitive consequences while walking. Neuropsychology. 2017;31(6):613–23. doi: 10.1037/neu0000331 28414497 PMC5578900

[15] Hsiu-Chen C, Chiung-Chu C, Jiunn-Woei L, Wei-Da C, Yi-Hsin W, Ya-Ju C, et al. The effects of dual-task in patients with Parkinson’s disease performing cognitive-motor paradigms. J Clin Neurosci. 2020;72:72–8. doi: 10.1016/j.jocn.2020.01.024 31952973

[16] Johansson H, Ekman U, Rennie L, Peterson DS, Leavy B, Franzén E. Dual-task effects during a motor-cognitive task in Parkinson’s disease: Patterns of prioritization and the influence of cognitive status. Neurorehabil Neural Repair. 2021;35(4):356–66. doi: 10.1177/1545968321999053 33719728 PMC8073879

[17] Broeder S, Nackaerts E, Nieuwboer A, Smits-Engelsman BCM, Swinnen SP, Heremans E. The effects of dual tasking on handwriting in patients with Parkinson’s disease. Neuroscience. 2014;263:193–202. doi: 10.1016/j.neuroscience.2014.01.019 24447597

[18] Criminger C, Swank C. Impact of dual-tasking on mobility tasks in Parkinson’s disease as described through 2D kinematic analysis. Aging Clin Exp Res. 2020;32(5):835–40. doi: 10.1007/s40520-019-01256-w 31270689

[19] Silva-Batista C, Corcos DM, Kanegusuku H, Piemonte MEP, Gobbi LTB, de Lima-Pardini AC, et al. Balance and fear of falling in subjects with Parkinson’s disease is improved after exercises with motor complexity. Gait Posture. 2018;61:90–7. doi: 10.1016/j.gaitpost.2017.12.027 29310015

[20] Yang Y-R, Cheng S-J, Lee Y-J, Liu Y-C, Wang R-Y. Cognitive and motor dual task gait training exerted specific training effects on dual task gait performance in individuals with Parkinson’s disease: A randomized controlled pilot study. PLoS One. 2019;14(6):e0218180. doi: 10.1371/journal.pone.0218180 31220121 PMC6586283

[21] Canning CG. The effect of directing attention during walking under dual-task conditions in Parkinson’s disease. Parkinsonism Relat Disord. 2005;11(2):95–9. doi: 10.1016/j.parkreldis.2004.09.006 15734667

[22] Kvist A, Peterson DS, Bezuidenhout L, Johansson H, Albrecht F, Ekman U. Exploring gait automaticity and prefrontal brain activity during single and dual-task walking in aging and Parkinson’s disease. J NeuroEngineering Rehabil. 2026;23(1):41. doi: 10.1186/s12984-025-01864-w PMC12849653 41491961

[23] Nieuwhof F, Reelick MF, Maidan I, Mirelman A, Hausdorff JM, Olde Rikkert MGM, et al. Measuring prefrontal cortical activity during dual task walking in patients with Parkinson’s disease: Feasibility of using a new portable fNIRS device. Pilot Feasibility Stud. 2016;2:59. doi: 10.1186/s40814-016-0099-2 27965875 PMC5154104

[24] Chan AW, Boutron I, Hopewell S, Moher D, Schulz KF, Collins GS, et al. SPIRIT 2025 statement: Updated guideline for protocols of randomised trials. BMJ. 2025;389:e081477. doi: 10.1136/bmj-2024-081477 PMC12035670 40294953

[25] Postuma RB, Berg D, Stern M, Poewe W, Olanow CW, Oertel W, et al. MDS clinical diagnostic criteria for Parkinson’s disease. Mov Disord. 2015;30(12):1591–601. doi: 10.1002/mds.26424 26474316

[26] Goodarzi Z, Mrklas KJ, Roberts DJ, Jette N, Pringsheim T, Holroyd-Leduc J. Detecting depression in Parkinson disease: A systematic review and meta-analysis. Neurology. 2016;87(4):426–37. doi: 10.1212/WNL.0000000000002898 27358339 PMC4977107

[27] Strouwen C, Molenaar EALM, Keus SHJ, Münks L, Munneke M, Vandenberghe W, et al. Protocol for a randomized comparison of integrated versus consecutive dual task practice in Parkinson’s disease: The DUALITY trial. BMC Neurol. 2014;14:61. doi: 10.1186/1471-2377-14-61 24674594 PMC3974198

[28] San Martín Valenzuela C, Moscardó LD, López-Pascual J, Serra-Añó P, Tomás JM. Effects of dual-task group training on gait, cognitive executive function, and quality of life in people with Parkinson disease: Results of randomized controlled DUALGAIT trial. Arch Phys Med Rehabil. 2020;101(11):1849-1856.e1. doi: 10.1016/j.apmr.2020.07.008 32795562

[29] Gaudet P. Measuring the impact of Parkinson’s disease: An occupational therapy perspective. Can J Occup Ther. 2002;69(2):104–13. doi: 10.1177/000841740206900206 11977869

[30] Kobayashi E, Himuro N, Mitani Y, Tsunashima T, Nomura K, Chiba S. Feasibility and informativeness of the Canadian occupational performance measure for identifying priorities in patients with Parkinson’s disease. Physiother Theory Pract. 2023;39(3):607–14. doi: 10.1080/09593985.2021.2023926 34986731

[31] Campos-Romero S, Lucchini Raies C, Rodríguez C, Muñoz P, Márquez-Doren F, Bustamante-Troncoso C. Adaptación cultural y validación del instrumento de Autoeficacia para el manejo de enfermedades crónicas en población chilena. Rev méd Chile. 2023;151(12):1596–603. doi: 10.4067/s0034-98872023001201596 39270081

[32] Clavijo M, Yévenes F, Gallardo I, Contreras AM, Santos C. Escala de autoeficacia general: Reevaluación de su evidencia de confiabilidad y validez en Chile. Rev Médica Chile. 2020;148(10):1452–60. doi: 10.4067/S0034-98872020001001452 33844715

[33] Bustamante-Contreras C, Ojeda-Gallardo Y, Rueda-Sanhueza C, Rossel PO, Martínez-Carrasco C. Spanish version of the mini-BESTest: A translation, transcultural adaptation and validation study in patients with Parkinson’s disease. Int J Rehabil Res. 2020;43(2):129–34. doi: 10.1097/MRR.0000000000000401 32097139

[34] Martínez-Martín P, Payo BF, Grupo Centro for Study of Movement Disorders. Quality of life in Parkinson’s disease: Validation study of the PDQ-39 Spanish version. J Neurol. 1998;245(Suppl 1):S34-8. 10.1007/pl00007737 9617722

[35] Dorsey ER, Elbaz A, Nichols E, Abbasi N, Abd-Allah F, Abdelalim A, et al. Global, regional, and national burden of Parkinson’s disease, 1990–2016: A systematic analysis for the Global Burden of Disease Study 2016. Lancet Neurol. 2018;17(11):939–53. doi: 10.1016/S1474-4422(18)30295-3 PMC6191528 30287051

[36] Leiva AM, Martínez-Sanguinetti MA, Troncoso-Pantoja C, Nazar G, Petermann-Rocha F, Celis-Morales C. Chile lidera el ranking latinoamericano de prevalencia de enfermedad de Parkinson. Rev Médica Chile. 2019;147(4):535–6. 10.4067/S0034-98872019000400535 31344222

