# Supplementary material for: The effects of dual task training to improve self-efficacy, functional performance, and quality of life in individuals with Parkinson’s disease: A study protocol
Source: PLoS One. 2026 Nov 12;21(11):e0362454. doi: 10.1371/journal.pone.0362454 (PMC13759104; doi:10.1371/journal.pone.0362454)
Supplement: Supplementary file 1 [file pone.0362454.s002.pdf]

PROPOSED RESEARCH

In this section, you are required to develop your proposal by addressing the following aspects: a) Theoretical-conceptual foundations and state of the art supporting the proposal, b) Research hypothesis or questions and objectives, c) Methodology, d) Work plan or Gantt chart, e) Background information demonstrating the capacity of the research team to carry out the proposed work and f) Scientific or technological novelty of the proposal.

Enhancing Self-Efficacy in individuals with Parkinson's Disease Through Dual Task Training: Effects of Task Modality, Attentional Prioritization, and Cognitive Status

The complex symptomatology of Parkinson´s disease and the role of Self-Efficacy in Rehabilitation

Parkinson´s disease (PD) is a chronic and progressive neurodegenerative disorder associated with early death of a specific group of neurons in the brain that regulates the neurotransmitter dopamine.[1–3] In individuals with PD, typical motor symptoms include resting tremor, rigidity, brady and hypokinesia.[4] As the disease advances, individuals experience freezing of gait and postural instability. [5,6] In addition to the more evident motor symptoms, people with PD also experience non-motor symptoms that include but are not limited to neuropsychiatric, gastrointestinal, autonomic, sleep, and sensory disturbances.[2,4,6–8] The heterogeneous nature of symptoms makes PD a complex disorder where motor and non-motor symptoms overlap and contribute to reduce physical, psychological and social wellbeing.[9–11]Individuals with PD experience restricted social participation, autonomy and self-efficacy in relation to healthy older adults that contribute to poor quality of life.[12] Self-efficacy is the perceived ability to perform specific tasks to achieve concrete goals successfully. [13] Individuals with higher self-efficacy usually have higher motivation, and self-care, improved health behavior, thinking pattern and emotional well-being.[11,14] Specifically in individuals with PD, lower self-efficacy is associated with worse motor and non-motor symptoms and lower quality of life.[15] In rehabilitation, an individual´s belief that she or he has the capacity to engage in exercise is positively associated with increased performance during rehabilitation and higher confidence in overcoming barriers to exercise.[16] For example, higher self-efficacy in a balance or cognitive task is associated with higher willingness to engage in rehabilitation activities and participation in the community. Self-efficacy and rehabilitation activities are reciprocally connected, so that an individual with high self-efficacy in performing a task will exhibit higher performance in the task which in turn will increase self-efficacy and motivation with the rehabilitation.[17]

Cognitive impairments in individuals with PD and its Functional Impact.

Cognitive impairment is a prevalent non-motor symptom in individuals with PD, exhibiting a range of manifestation and varying degrees of severity.[18] Cognitive impairments affect from 20% to 57% of individuals with PD, and they may be present years before a diagnosis of PD is made.[19,20] From the early to late stages of the disease, cognitive impairments progress from mild cognitive impairments to dementia.[21] Memory, executive control, visuo-spatial abilities, processing speed and attention are the most affected cognitive domains in individuals with PD.[20] Studies in older adults and individuals with neurodegenerative diseases reveal a strong association between cognitive deficits, gait impairments, and fall risk.[22] Specifically in PD, a prospective study by Wood et al. (2002) demonstrated that individuals with PD and dementia faced a significantly higher risk of falling compared to those without dementia.[23] Traditionally, PD is considered a movement disorder; however, cognitive impairments may have similar or greater impact in reducing self-efficacy, functional independence, social engagement, and overall quality of life.[23,24] Mostly, cognitive and motor problems in PD are examined and treated separately, but the impact that the interaction of motor and cognitive problems produces in individuals with PD, strongly suggests that an integrated care approach is necessary to improve motor and cognitive self-efficacy and ultimate quality of life of individuals with PD and their families.[25]

Attentional and executive function deficits in individuals with PD.

The degeneration of dopaminergic cells in individuals with PD leads to a progressive decline in their ability to control automatic movements involved in day-to-day activities (e.g. waking, talking, picking up a cup to drink).[26,27] Consequently, individuals with PD when performing two tasks simultaneously (one motor and one cognitive or two motor tasks) have fewer attentional resources to perform the secondary task.[28] For example, it may be that an individual fails in memorizing a shopping list and pays more attention to posture and stability to avoid falling while shopping or stops walking while holding a cup of tea. Attention is a limited resource, and dividing attention between two tasks performed concurrently can negatively impact performance in one or both tasks.[29] This phenomenon, known as dual-task interference or dual-task effect, refers to the change in performance when performing two tasks (dual tasking) compared to performing a single task (single tasking).[30,31] Under dual tasking, studies reveal that individuals with PD exhibit reduced balance control,[32,33] decreased gait speed, stride length, and cadence,[34–36] reduced cognitive performance (executive function, spatial memory, calculation and reaction time),[37–39] reduced speed and amplitude during hand writing [40] and reduced mobility.[41] Furthermore, individuals with PD and mild cognitive impairments demonstrate greater dual-task interference than those without cognitive impairments.[39] Individuals with PD may then avoid dividing attention between simultaneous tasks during daily activities for fear of falling or embarrassment in a cognitive task. Precisely, higher fear of falling has revealed to be associated with higher cognitive

impairments.[42] This reduced self-efficacy in performing dual tasks becomes a self-fulfilling prophecy because fear and lack of confidence reduce the willingness and motivation for becoming more physically active and participative in the community. Therefore, targeted interventions focused on motor cognitive training seem crucial to overcome disease-related barriers and improve self-efficacy.

Motor-cognitive (dual tasking) training modalities in individuals with PD.

Motor-cognitive training can be conducted either sequentially or simultaneously. In sequential motor training, each training component is performed separately, one after the other (e.g., stationary cycling followed by an arithmetic task or vice versa). On the other hand, simultaneous motor-cognitive training involves performing both (motor and cognitive) tasks concurrently.[43,44] Furthermore, dual task training can also involve performing two motor tasks simultaneously such as walking and bouncing a ball (motor dual tasking). Most of the studies in individuals with neurological diseases and in PD have implemented motor-cognitive dual tasking training instead of motor dual tasking training programs. In individuals with PD, it seems that the dual task cost or interference in performing two tasks is higher during motor-cognitive dual tasking than in motor dual tasking. This suggests that motor-cognitive dual tasking may be more challenging.[45] Which dual tasking training modality is more beneficial in individuals with PD with and without cognitive impairments is unknown. Whether a secondary cognitive or motor task produces more interference on a primary motor task depending on the cognitive status of individuals with PD has not been studied. It is unclear if greater interference of a secondary task is associated with greater benefits after dual tasking training.

Attention and prioritization instructions during dual task training.

During dual tasking, individuals can be instructed to direct the attention to both tasks (fixed priority instructions) or to shift attention between tasks (variable priority instructions). Evidence indicates that motor performance deteriorates when individuals with PD are instructed to pay equal attention to both tasks, and it improves when they are instructed to prioritize the primary over the secondary task.[46] Furthermore, the instruction of attentional allocation may have different effects on individuals with PD depending on their cognitive level. Research suggests that individuals with PD and mild cognitive impairment tend to prioritize cognitive tasks over posture, whereas those with PD and no cognitive impairment prioritize posture over cognitive tasks.[39] This prioritization strategy may increase the risk of falls in PD patients with cognitive impairments, particularly in cases when prioritization instructions are not explicit. The more beneficial strategy for individuals with PD and cognitive impairments also remains unclear.

Patient reported outcomes measures.

Functional and daily living impairments in individuals with PD will depend not only on the severity and variety of symptoms (unique combination of signs and symptoms) but also on personal (e.g. coping mechanisms, attitude, motivation) and environmental factors (e.g. culture, socio economic).[47] In consequence, a patient centered approach incorporates what outcome is important or meaningful for them to improve after participating in a clinical trial. The incorporation of patient reported outcomes during the rehabilitation process have demonstrated multiple benefits and it is considered a cornerstone of contemporary practice in rehabilitation. A patient reported outcome is an information about the health condition or status of participants that is provided directly by the participant without interpretation of the researcher or clinician.[48,49] **Patient reported outcomes are complementary to clinical and biomedical outcomes and have the potential to improve engagement of participants in clinical trials.** [50,51] This approach is patient centered and may be more useful for assessing the impact of dual tasking interventions on functionality, self-efficacy and quality of life. However, patient reported outcomes are subjective and may not necessarily have a correlation with clinical or physiological markers of PD. Therefore, it seems necessary to include subjective and “objective” outcomes and compare their agreement after a dual tasking training in individuals with PD.

Brain imaging techniques

Functional near-infrared spectroscopy (fNIRS) and electroencephalogram (EEG) are useful brain imaging techniques that allow continuous recordings of brain activity in real time while performing single and dual tasks activities.[52] Functional magnetic resonance (FMRI) and positron emission tomography (PET) are also valid and reliable methods for the same purpose; however, the utility of these last two neuroimaging techniques is limited because participants must imagine or imitate the task. This shortcoming naturally reduces the generalization of the results to actual and real time dual tasking activities.[53] Compared with EEG, fNIRS has become more useful for cognitive tasks because fNIRS has shown to be resistant to motion artifacts, and more precise for measuring brain activity during dual tasking activities.[54]

Functional near-infrared spectroscopy (fNIRS) during dual tasking

The dorso and ventro lateral prefrontal cortex exhibits greater activation during dual tasking activities when compared with single task conditions.[55] This higher neuronal activation is accompanied with higher blood supply to satisfy the increased metabolic demand of this area of the brain. The increased oxygen metabolism produces greater oxygen demand and concentration of oxygenated hemoglobin as well as reduced concentration of deoxygenated hemoglobin in the blood vessels.[56] Functional near-infrared spectroscopy (fNIRS) is an optical neuroimaging technique used to detect changes in

oxygenated and deoxygenated hemoglobin levels in brain areas and therefore changes related to higher or lower neuronal activity.[57] Given that individuals with PD have reduced capacity to perform automatic movements, they are forced to use extra attentional resources of the prefrontal cortex to perform dual tasking activities safely. Therefore, in individuals with PD, higher neuronal activity in the prefrontal cortex during dual tasking indicates increased oxygen metabolism and demand, over-recruitment of this brain area, and reduced efficiency in executing dual tasks.[58] Functional NIRS has demonstrated to be feasible, well tolerated and sensitive technique to measure neuronal activity of the prefrontal cortex during dual tasking in individuals with PD.[59] **The main advantages of the fNIRS are that it is not invasive, portable, sensitive and able to measure cortical activity in real time during the actual performance of dual tasking activities, providing more ecologically valid results.** Examining neuronal activity of the prefrontal cortex (dorso and ventro lateral) in individuals with and without cognitive impairments under single and dual task conditions will provide a better understanding of physiological changes of the brain of individuals with PD.

In summary, motor and cognitive symptoms are debilitating problems in individuals with PD and have an important impact in reducing self-efficacy, functional independence, social engagement, and overall quality of life. Besides, motor and cognitive problems as well as mood disorders have a negative and bidirectional association with self-efficacy so that fear of falling and depressive symptoms contribute to low self-efficacy and lack of self-efficacy lead to depressive symptoms, and reduced motivation in engaging in physical and social activities. The neuropathological changes caused by the degeneration of dopaminergic neurons affect the ability to control automatic movements. Therefore, when performing two tasks simultaneously (one motor and one cognitive or two motor tasks) individuals with PD must activate extra brain regions and allocate extra attentional resources. Attention is a limited resource, and dividing attention between two tasks performed concurrently can negatively impact the performance in one or both tasks. This reduced attention capacity has a negative impact in reducing self-efficacy. Therefore, interventions focused on motor cognitive training are relevant to overcome disease-related barriers and improve self-efficacy. However, different dual tasking modalities may have a dissimilar beneficial effect in self-efficacy depending on the cognitive level of individuals with PD. In addition, the incorporation of patient reported outcomes are important to have a multidimensional assessment of the impact of dual tasking interventions in self-efficacy. In this research proposal our purpose is to compare different dual task training modalities and determine whether motor dual tasking or motor cognitive dual tasking training as well as dual tasking with variable or fixed priority instructions is more beneficial for improving self-efficacy in individuals with PD. Particularly, we want to know which modality is more beneficial for improving self-efficacy depending on the cognitive status of individuals with PD. In addition, our purpose is to determine whether improvements in self-efficacy are associated with changes in motor and cognitive function, cortical activity and patient reported outcomes. To address these aims this research proposal includes two studies. One is an experimental study in individuals with PD with and without cognitive impairments. The second study is a cross-sectional study that includes healthy controls and the same individuals with PD measured at baseline for study 1. **In this research proposal, an interdisciplinary group of professionals (including neurologists, physical therapists, occupational therapists and psychologists) will design and implement different dual task training interventions for individuals with PD with and without cognitive impairments based on individual baseline measurements and patient reported outcomes so that are relevant and meaningful for participants and their functional performance during activities of daily living.**

Objectives and hypothesis

General objective

To investigate the effects of dual task training in improving self-efficacy, functional performance and quality of life and to analyze the association of these improvements with changes in motor-cognitive function and brain activity of the prefrontal cortex in individuals with Parkinson's disease with and without cognitive impairments.

General hypothesis

The general hypothesis is that dual task training will lead to significant improvements in self-efficacy, functional performance and quality of life. These improvements will be associated with greater motor-cognitive function and reduced brain activity of the prefrontal cortex during dual task performance, indicating increased neural efficiency. These effects are expected to occur regardless of the participants' cognitive status.

Specific objective 1: To identify whether motor dual task training (two motor tasks) or motor cognitive training (primary motor and secondary cognitive task) either one combined with variable or fixed priority instructions has greater benefits in improving self-efficacy, functional performance and quality of life according to the cognitive level of individuals with Parkinson's disease.
Specific hypothesis 1: Dual task training that incorporates a secondary cognitive task combined with variable prioritization instructions will produce greater improvements in self-efficacy, functional performance, and quality of life, as well as a more pronounced reduction in prefrontal cortex activity during dual-task performance, compared to training that uses a secondary

motor task with fixed prioritization instructions. These benefits are expected to be more substantial in individuals with cognitive impairments than in those without, indicating a differential effect based on cognitive status.

Specific objective 2: To determine whether the amount of interference (dual task cost) produced across dual task training modalities is associated with greater self-efficacy, functional performance and quality of life in individuals with Parkinson's disease with and without cognitive impairments.
Specific hypothesis 2: Higher levels of dual-task interference (i.e., greater dual-task cost) will be associated with greater improvements in self-efficacy, functional performance, and quality of life following dual-task training in individuals with Parkinson's disease, regardless of cognitive status. This suggests that individuals who initially exhibit greater difficulty under dual-task conditions may derive the most benefit from targeted intervention.

Specific objective 3: To compare the amount of interference (dual task cost) and brain activity in the prefrontal cortex produced under dual tasking in healthy controls and individuals with Parkinson's disease. In this cross-sectional study, dual task cost and brain activity will be measured in individuals with Parkinson's disease and healthy controls while performing motor dual tasking (two motor tasks), motor cognitive dual tasking (primary motor and a secondary cognitive task) either one combined with fixed or no priority instructions (equally divided attention) (4 conditions).
Specific hypothesis 3: Motor-cognitive dual-tasking performed under conditions of equally divided attention (i.e., without prioritization instructions) will elicit greater dual-task interference and increased activation of the prefrontal cortex compared to motor-motor dual-tasking with fixed prioritization instructions. This effect is expected to be observed in both healthy controls and individuals with Parkinson's disease, reflecting higher cognitive demand and reduced neural efficiency under non-prioritized conditions.

Expected Results

It is anticipated that dual task training using a secondary cognitive tasks and variable prioritization instructions is more beneficial for improving self-efficacy, functional performance and quality of life than dual tasking training using secondary motor tasks with fixed prioritization instructions. The rationale behind this hypothesis is that activities of daily living often require shifting task prioritization and attention, which aligns better with the variable prioritization modality. Additionally, variable priority instructions better simulate real-life situations, making the training more meaningful and ecologically valid for participants. Consequently, the benefits obtained from dual-task training with variable prioritization are anticipated to be more transferable to real-life situations. Furthermore, it is anticipated that individuals with cognitive impairments will benefit more from motor-cognitive training, as it may provide additional cognitive support compared to a dual-task motor training program. It is expected that the greater the interference during dual tasking and after dual task training, the greater the benefit in self-efficacy, functional performance and quality of life. These findings will contribute to the design of specific and tailored dual-task training programs for individuals with PD with or without cognitive impairments. Lastly, it is expected that brain activity in the prefrontal cortex will be a physiological marker of interference under dual tasking in individuals with Parkinson's disease.

Scientific novelty and impact

Parkinson's disease is the fastest growing neurological disorder in prevalence, disability and death worldwide. [60] Chile is the Latin American country with the highest rise in PD prevalence and aging is considered a significant contributor to the increasing prevalence of the disease.[60,61] To date there is no cure for PD and therefore there is a growing demand in the PD community for accessing person centered therapeutic interventions that have an impact in improving quality of life. Parkinson's disease entails progressive loss of autonomy, often leading to learned helplessness, anxiety, and depression Despite optimal medication management, motor and non-motor symptoms progressively impair functional independence and self-efficacy. Diminished self-efficacy is associated with reduced engagement in physical activity, avoidance of complex or novel tasks, and heightened fear of falling; factors that exacerbate physical decline and social isolation. Targeting self-efficacy within rehabilitation programs enhances participants' perceived control over their condition, improves coping mechanisms, and promotes resilience. Therefore, self-efficacy becomes a critical determinant of long-term psychological adjustment and quality of life. However, there are no proven strategies to improve self-efficacy in individuals with PD. Dual tasking interventions have the potential to improve motor and cognitive function with a positive impact in improving self-efficacy. With the proposed research, our goal is to provide person centered, enjoyable and tailored dual task training that has a significant clinical impact on self-efficacy and quality of life in individuals with PD and their caregivers.

Finally, the neurophysiological mechanisms underlying dual-task interference in individuals with PD remain insufficiently characterized. While dual-task training modalities are used to improve motor cognitive function in individuals PD, the specific

patterns of brain activity associated with distinct dual-task modalities are unknown. This knowledge gap is particularly pronounced in individuals with PD and cognitive impairments, in whom dual-task performance is markedly reduced. Critically, it remains unknown whether alterations in brain activity during dual tasking are associated with clinically meaningful outcomes such as self-efficacy. Advancing the understanding of the neural correlates of dual-task costs is essential to uncover the underlying neurophysiological substrates of cognitive-motor interference in PD. Such insights could inform the development of targeted neurorehabilitation strategies and serve as potential biomarkers for disease progression and intervention efficacy.

Methodology

Ethical considerations

Participants in this research project will receive comprehensive documentation and information regarding the study. The researchers will ensure that participants provide voluntary consent before entering the study, and participants will have the option to withdraw at any time without facing any consequences. The involvement of participants with cognitive impairments will consider additional safeguards, such as the use of advanced informed consent, the participation of a legal representative, surrogate consent and assent. The risks associated with dual tasking testing/training are minimal, although participants may experience fatigue during the assessments. To mitigate this, all measurements will be conducted while participants are in the "on" state of medication, and appropriate resting periods will be scheduled between measurements. Additionally, all training sessions will be supervised by experienced therapists who will continuously monitor the participants' well-being. It is worth noting that dual task training has proven to be safe and well-tolerated in individuals with neurodegenerative disorders. Therefore, the experimental design of this research proposal will not impose an excessive or unequal burden on the participants involved in this research project. All research procedures will be conducted in accordance with the Declaration of Helsinki. This research project and informed consent will be submitted to our institutional Ethical Committee.

Study 1

Experimental design

This study will employ a (2x2) factorial experimental design nested in a factor variable (cognitive impairment) as depicted in the Figure 1 below (nested factorial design). Participants with PD will be allocated to each group using the block randomization method. Allocation concealment will be implemented using sequential sealed envelopes prepared by a research assistant independent from the research team. **The intervention will be delivered individually to each participant by at least two trained therapists with experience in dual tasking training**. The therapists will be specifically dedicated to providing the interventions. **Therapists will provide dual task training following the planification provided by the interdisciplinary group and their assessments performed at baseline.** The research assistant will notify the therapists by emailing the group allocation to ensure concealment. Data will be collected by researchers blinded to group allocation. All participants will be measured at baseline and after 24 sessions of dual tasking training.

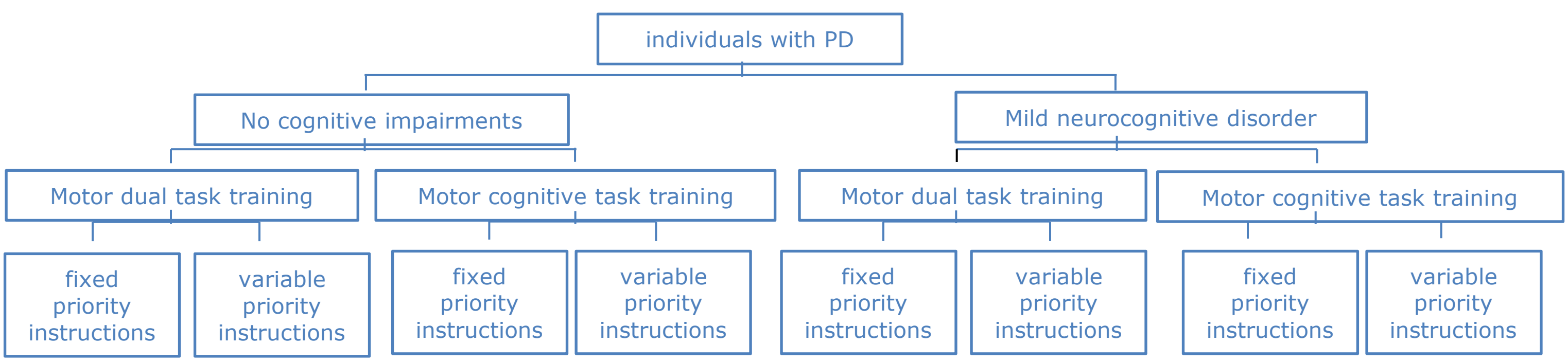

Figure 1. Factorial experimental design

Participants

Participants diagnosed with idiopathic Parkinson's disease confirmed by a neurologist will be recruited in this study. Inclusion criteria will be: 1) Hoehn & Yahr stages I, II or III, 2) participants with no or mild to moderate neurocognitive impairments, 3) participants with and with-out symptoms of depression, 4) able to walk independently, 5) unaltered dose and type of medication up to three months before the commencement of the study, 6) participants able to understand and respond to the instructions given in the study. Exclusion criteria will be: 1) the presence of any other concomitant neurological, musculoskeletal disease, 2) history of leg or brain trauma or surgery, 3) vision or hearing problems that may affect the performance of cognitive tests, and 4) clinical diagnosis of major neurocognitive disorder. Neurocognitive disorder will be determined by the criterion defined by *The Diagnostic and Statistical Manual of Mental Disorders, Fifth Edition, Text Revision*

(DSM-5 -TR) previously diagnosed by a specialist. The 15-item Geriatric Depression Scale will be used for depression symptoms screening because it has shown the highest diagnostic sensitivity.[62] This study will be submitted to the institutional Ethical Committee. Informed written consent will be obtained from all participants before baseline measurements.

Sample size calculation
The studies by Strouwen et al., 2017 and Valenzuela et. al., 2020 were consulted for sample size because the authors reported the dual-task cost of motor tasks while performing cognitive tasks and the dual task cost of cognitive task while performing motor tasks (dual task cost of both tasks). We anticipate that there will be a difference of at least 12% between dual tasking training modality. From the results of these studies, we aim to estimate a large effect (Cohen's f=0,40) for gait velocity under dual task after the intervention. Statistical power was set at 0.8 and the alpha level at 0.05 with two tails. For a 2x2 factorial experimental design nested in the cognitive status of participants, to examine the effects of dual task training modality, priority instructions, time (pre post intervention) and interactions it was estimated a sample size of at least 12 participants per group (8 groups) and 96 in total. We also estimate a drop-out rate of 13% per group. Therefore, we aim to recruit 14 participants per group and 112 in total over a period of 2.5 years. To achieve the required sample size, we have stablished collaborations with institutions that have expressed their support and willingness to collaborate in disseminating and recruiting participants for this research proposal (please see support letters included in the supplementary section of this application).

Procedures
The purpose and nature of the study will be explained to all individuals interested in the study. Written informed consent will be obtained from all participants who fulfilled the inclusion criteria. Before the commencement of the intervention, all participants will be interviewed regarding their general health status. Sociodemographic data and information regarding each participant's Parkinson´s disease will be recorded. All outcomes will be measured before and after 24 sessions of dual tasking training by researchers blinded to group allocation. **Baseline measurement will be performed by the interdisciplinary group of physical therapist, occupational therapist and psychologist.** Participants will be familiarized with the assessments before data collection. After familiarization, cognitive and motor functions under single and dual-task conditions will be repeated three times to assess the test-retest reliability. Before data collection, the interdisciplinary team will perform rehearsal testing sessions for all the assessments to standardize the procedures, criteria, and results' interpretation and ensure data quality. Assessments and interventions will be conducted with participants in the "on" state of medication. All measurements and intervention will be performed in a dedicated laboratory at our university.

Intervention (dual task training)
All participants will receive 24 sessions of dual task training, 3 sessions per week, for 8 weeks for 60 minutes each session. **Dual task training will be delivered individually to each participant. Therapists will deliver dual-task training based on the assessments and program individually designed by the interdisciplinary team at baseline.** Participants in the motor-cognitive task training will be instructed to perform motor tasks while simultaneously performing cognitive tasks. Examples of motor cognitive tasks are walking or balancing while spelling words forward and backwards, walking/balancing while naming objects starting with a specific letter, walking/balancing, and summing and counting or walking/balancing while doing go no go tasks. Cognitive tasks include tasks related to attention, working memory, inhibition, flexibility, perceptual performance, mental tracking, reaction time and executive performance. Motor tasks include walking forward or backwards, walking in tandem and walking on an S-shaped route, transfers from sitting to standing, rolling from supine to sitting and from sitting to supine. Participants in the motor dual task training will be instructed to perform two motor tasks simultaneously. Examples of dual motor tasks are walking while holding a tray with or without glasses, walking while bouncing balls of different diameters, walking while throwing and catching a ball. Motor-cognitive training will include activities of daily living and functional performance of occupations thar are meaningful and relevant for each person. Participants under variable priority instructions will be trained to focus attention on walking performance during half of the training session and in the rest of the session to focus on the performance of the secondary cognitive or motor task depending on group allocation. Participants under variable priority instructions will be trained to maintain attention on both tasks (motor and cognitive or motor and motor) through the training session. **In all groups, dual task exercises will be standardized as well as the progression and the difficulty of both tasks. To design dual task training, motor and cognitive tasks will be selected and adapted based on baseline measurements, functional necessities of each participant and patient centered goals. Participants will progress to a more difficult dual task once they can perform the task fluently or without evident dual-tasking interference. Dual task training interventions will incorporate important parameters for experience-dependent neuroplasticity that include intensity, feedback, repetition, specificity, difficulty and variability**.

Measurements

Self-efficacy

The Latin Spanish version of the Self-Efficacy to Manage Chronic Disease for the Chilean population (SEMCD-S)[63] and the General Self-Efficacy scale (GSES) [64] will be used for this outcome. In the SEMCD-S participants rank each one of the 4 items from 0 to 10. Higher scores indicate higher self-efficacy. In the GSES participants rank each one of the 10 items from 0 to 7. Similarly, higher scores indicate higher self-efficacy. Both instruments have revealed to be useful, valid and reliable with validated versions in Spanish.

Unified Parkinson's Disease Rating Scale

The sections II and III of the Unified Parkinson's Disease Rating Scale will be measured to analyse the effect of the intervention on activities of daily life and clinical motor aspects of the disease.

Balance

The Mini BesTest will be used to assess static and dynamic balance during functional activities. The Mini BesTest has demonstrated to be valid and reliable clinical measure for assessing balance in individuals with Parkinson's disease. The Mini BesTest has also been validated in Chile.[65]

Gait

Spatiotemporal gait parameters under single and dual-task conditions will be measured using a pressure-sensitive mat (zebris FDM-2® Medical GmbH, Germany). During the single-task condition, participants will be asked to walk at a self-selected walking speed covering 10m. Under dual-task condition, participants will be asked to perform the same as above while performing cognitive tasks. Walking speed (m/s), cadence (steps/min) and stride length (cm) will be recorded for analysis.

Cognitive functions

The Serial Threes Test (STT) and the auditory Stroop task will be used to evaluate cognitive functions under single and dual-task conditions. The STT requires participants to make multiple subtractions of three from a whole three-digit number, *e.g.*, 256, 253, 250, 247. Different three-digit numbers will be used for each trial to reduce the learning effect. The auditory Stroop task require participants to verbally respond to congruent and incongruent high and low tones. Participants will hear the words "high" and "low" that were recorded in either a high or low pitch. Participants will be asked to ignore the actual word presented and indicate the pitch of the word they heard. The Serial Threes Test (STT) and the auditory Stroop task will be used because they are demonstrated to be sensitive to detect changes as well as feasible, reliable, and valid during dual tasking in individuals with PD.[59,66,67]

Patient Reported Outcome Measure

The Canadian Occupational Performance Measure will be administered as patient reported outcome measure. This is a personalized outcome assessment tool that utilizes a semi-structured interview along with a structured scoring system. During the interview, participants select three to five activities they find challenging and wish to improve. Participants rate these activities on a scale from 1 to 10, evaluating both their perceived performance ability and their satisfaction with it. In individuals with Parkinson's disease the Canadian Occupational Performance Measure has demonstrated to be useful, valid and reliable.[68,69] This instrument has an official and validated version in Spanish.

Quality of Life

Quality of life will be measured using the Spanish version of the Parkinson´s disease questionnaire (PDQ-39). This instrument includes 39 questions distributed in eight domains where 0% is no problem and 100% is maximum level of problem.[70] This instrument has an official version for Chilean population.

Brain activity of the Prefrontal cortex

The hemodynamic activity of the dorso and ventrolateral prefrontal cortex will be measured using a functional Near-Infrared Spectroscopy (fNIRS Brite Artinis, The Netherlands) system under single and dual-task conditions. A head cap containing two light emitters and detectors (devices) will be placed on the scalp on the right and left side of the forehead of each participant. In the sagittal plane, both detectors will be placed at a distance of 15% of the length of the nasion-inion starting from the nasion. In the transverse plane each detector will be placed 7% of the head circumference starting from the midline. The devices will be placed on these scalp areas to detect brain activity of the prefrontal cortex (left and right Brodmann`s areas 9 and 10). The devices will be protected against ambient light using a black cloth covering the entire forehead. Raw

data will be converted to relative changes in oxygenated and deoxygenated haemoglobin concentrations using the modified Lambert-Beer law. During testing, the blood pressure and heart rate will be monitored continuously on each participant to control for a systemic response.

Dual task cost
Motor and cognitive outcomes will be measured under single and dual-task conditions. Under single cognitive task individuals will be asked to perform two tasks: the serial threes test (STT) and the auditory Stroop task. The number of correct responses will be recorded for analysis in both tasks. For single motor task, individuals with PD will be instructed to walk at a self-selected speed covering 10m. Walking speed (m/s) will be recorded for analysis. Under dual tasking, the motor task will be combined with a secondary cognitive task (serial threes test and the auditory Stroop task) and a secondary motor task (holding a tray with glasses and bouncing a ball) to be executed simultaneously. Single and dual tasks will be performed randomly to prevent the learning effect. Single and dual tasks will be repeated three times to assess test-retest reliability. The average of three trails will be calculated for analysis. Brain activity of the prefrontal cortex will be measured using a functional Near-Infrared Spectroscopy (fNIRS) system under single and dual-task conditions. Dual-task cost values will be calculated to compare the performance on dual task modality using the following formula:

$$dual\ task\ cost(\%) = \frac{dual\ task\ performance - single\ task\ performance}{single\ task\ performance} x100$$

A negative value indicate that the performance deteriorates in dual task condition relative to single task condition. A positive value indicates a relative improvement in dual task in relation to the single task condition.

Statistical Analysis
Descriptive data will be given as mean and standard deviation. A one-way analysis of variance and a Scheffé post hoc test will be performed to compare physical and physiological characteristics and baseline measurements between groups. To assess the test-retest reliability of motor and cognitive tasks under single and dual-task conditions, the interclass correlation coefficient and the coefficient of variation will be calculated for each group. Normality assumption will be tested using the Shapiro-Wilk test. Changes in outcomes measures after the intervention will be tested using a linear mixed model. The model will include self-efficacy, motor and cognitive function outcomes, quality of life, brain activity of the prefrontal cortex as response variable. Time (pre versus post intervention), group and interaction (group x time) will be fitted as explanatory variables. Group will be a fixed effect factor and time a random effect factor. Changes in patient reported outcomes will be analyzed using the Friedman's and Wilcoxon rank-sum tests. Correlation between the amount of interference (dual task cost), patient reported outcomes and brain activity will be assessed using the Pearson and Spearman correlation coefficients. Statistical significance will be set at $P \leq 0.05$. Statistical analyses will be performed using STATA statistical software version 18.0 and R version 4.3.2.

Study 2
Experimental design
This study will employ a cross-sectional design that will include the group of participants with PD in study 1 and another group of healthy volunteers matched by age, gender, and educational level. The same measurements performed on individuals with PD at baseline in study 1 will be performed on the group of healthy controls. All assessments will be performed by trained therapists.

Participants
For individuals with PD, the same characteristics, inclusion, and exclusion criteria described in study 1 will apply in study 2. For healthy controls inclusion criteria will be: 1) volunteers matched by age, gender, and educational level, 2) able to walk independently. Exclusion criteria will be: 1) the presence of any other concomitant neurological, cardiovascular, or musculoskeletal disease that interferes with walking 2) hearing problems. Informed written consent will be obtained from healthy controls before the baseline measurements.

Sample size calculation
Oxygenated hemoglobin under dual-task conditions will be the primary outcome of this experiment. The studies by Maidan et al., 2017 and Nieuwhof et. al., 2016 were consulted for sample size. From the results of these studies, we estimate a medium effect size (Cohen's f=0,25) between groups (healthy controls, PD with no cognitive impairments, PD with cognitive impairments). For a one-way analysis of variance (ANOVA), with an alpha level of 0.05, a statistical power of 0.8, it was

estimated a sample size of at least 53 participants per group. Given that for study 1 we expect to recruit 56 participants with PD and cognitive impairments and 56 participants with PD and no cognitive impairments, we will recruit 56 healthy controls.

Procedures

The same procedures described in experiment 1 for individuals with PD at baseline will be performed in healthy controls.

Measurements

The same measurements described in experiment 1 for individuals with PD at baseline will be performed in healthy controls.

Statistical Analysis

Descriptive data will be given as mean and standard deviation. To assess the test-retest reliability of motor and cognitive tasks under single and dual-task conditions, the interclass correlation coefficient and the coefficient of variation will be calculated for each group. Normality assumption will be tested using the Shapiro-Wilk test. Differences in dual task cost and brain activity of the prefrontal cortex due to group factor will be tested using a one-way analysis of variance ANOVA and a Scheffé post hoc test. Statistical significance will be set at $P \leq 0.05$. Statistical analyses will be performed using STATA statistical software version 18.

Contingency Plan

If participant recruitment and sample size for Study 1 is under expected, a comprehensive contingency plan will be implemented to mitigate this risk. This plan includes the establishment of additional collaborations with clinics and hospitals to expand the recruitment network. Furthermore, we will strengthen partnerships with PD associations and support groups to facilitate broader dissemination of the study within the community of individuals with PD. To enhance dissemination, the study will be promoted through digital platforms, including social media, institutional websites, and online forums, thereby increasing visibility among potential participants and caregivers. The project timeline, as outlined in the Gantt chart, allows for continuous and flexible recruitment over an extended period, rather than relying on fixed enrollment windows. This approach is designed to maximize recruitment opportunities and accommodate participant availability. To reduce barriers to participation, the study will offer transportation stipends, flexible scheduling options, and follow-up support. Regular communication will be maintained with participants through reminder calls and messages, along with periodic updates on study progress to foster engagement and retention. Importantly, any such modifications will be communicated promptly to the funding agency and the institutional ethics committee. Formal amendments will be submitted as required to ensure full compliance with ethical standards.

Background information

We have an outstanding research team that includes national and international clinicians and researchers with strong reputation in neurorehabilitation and extensive experience in designing and conducting clinical trials. In addition, the research team is strengthened by international collaborators. Dr. Travis Cruickshank, from the Centre for Precision Health at Edith Cowan University (Perth, Western Australia), brings specialized expertise as a neuroscientist and exercise scientist with a strong track record in designing and evaluating motor-cognitive interventions for individuals with neurological disorders. Together, this collaborative and interdisciplinary framework positions the research team to deliver a scientifically robust and clinically impactful investigation. We have a strong support from the biggest community in Parkinson' disease in Chile to ensure the feasibility of this investigation. The objective of this proposal targets high priority needs of individuals living with Parkinson's disease. This study has the very real potential to provide ground-breaking evidence to support an intervention that changes the trajectory of the disease and improves quality of life in individuals with Parkinson's disease, families, and caregivers. Our results will also provide strong evidence for the development of national and international guidelines for the treatment of Parkinson's disease. This intervention may also be broadly relevant to other neurodegenerative conditions where treatments are also urgently needed.

The work plan of this research proposal is presented in the following Gantt chart:

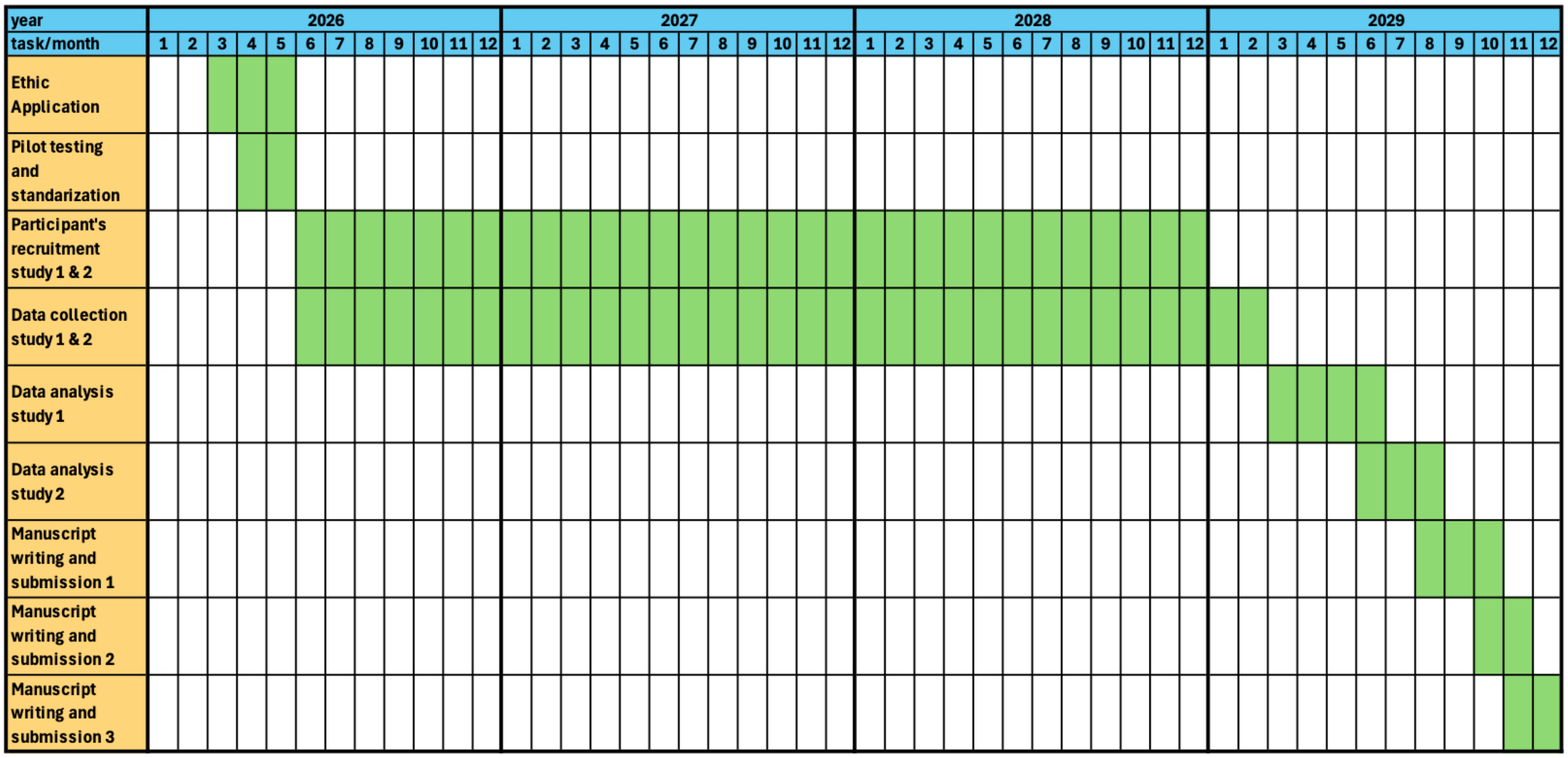

References

1. Radder DLM, Lígia Silva de Lima A, Domingos J, et al. Physiotherapy in Parkinson's disease: a meta-analysis of present treatment modalities. *Neurorehabil Neural Repair* 2020;34(10):871–880.
2. Vijiaratnam N, Simuni T, Bandmann O, Morris HR, Foltynie T. Progress towards therapies for disease modification in Parkinson's disease. *Lancet Neurol* 2021;20(7):559–572.
3. Kalia L V, Lang AE. Parkinson's disease. *Lancet* [Internet] 2015;386(9996):896–912. Available from: http://www.ncbi.nlm.nih.gov/pubmed/25904081
4. Tysnes O-B, Storstein A. Epidemiology of Parkinson's disease. *J Neural Transm* 2017;124:901–905.
5. Conradsson D, Löfgren N, Nero H, et al. The effects of highly challenging balance training in elderly with Parkinson's disease: a randomized controlled trial. *Neurorehabil Neural Repair* 2015;29(9):827–836.
6. Samii A, Nutt JG, Ransom BR. Parkinson's disease. *The Lancet* 2004;363(9423):1783–1793.
7. Reich SG, Savitt JM. Parkinson disease. *Medical Clinics of North America* 2018;
8. Chaudhuri KR, Healy DG, Schapira AH V. Non-motor symptoms of Parkinson's disease: diagnosis and management. *Lancet Neurol* 2006;5(3):235–245.
9. Rajan R, Brennan L, Bloem BR, et al. Integrated care in Parkinson's disease: a systematic review and meta-analysis. *Movement Disorders* 2020;35(9):1509–1531.
10. Giladi N, Manor Y, Hilel A, Gurevich T. Interdisciplinary teamwork for the treatment of people with Parkinson's disease and their families. *Curr Neurol Neurosci Rep* 2014;14:1–7.
11. Tinaz S, Elfil M, Kamel S, Aravala SS, Louis ED, Sinha R. Goal-directed behavior in individuals with mild Parkinson's disease: Role of self-efficacy and self-regulation. *Clin Park Relat Disord* 2020;3:100051.
12. Kim MY, Jethani P, Kang E, Lipsey KL, Foster ER. Conceptualization, measurement, and factors associated with participation in Parkinson disease: A systematic review and qualitative analysis. *Arch Phys Med Rehabil* 2024;105(6):1181–1193.
13. Bandura A. Self-efficacy: The exercise of control. Macmillan; 1997.
14. Ritter PL, Lorig K. The English and Spanish self-efficacy to manage chronic disease scale measures were validated using multiple studies. *J Clin Epidemiol* 2014;67(11):1265–1273.
15. Estrada-Bellmann I, Meléndez-Flores JD, Cámara-Lemarroy CR, Castillo-Torres SA. Determinants of self-efficacy in patients with Parkinson's disease. *Arq Neuropsiquiatr* 2021;79(8):686–691.
16. Stevens A, Stanton R, Rebar AL. Helping people with Parkinson disease build exercise self-efficacy. *Phys Ther* 2020;100(2):205–208.
17. Ehlers DK, Banducci SE, Daugherty AM, et al. Effects of Gait Self-Efficacy and Lower-Extremity Physical Function on Dual-Task Performance in Older Adults. *Biomed Res Int* 2017;2017(1):8570960.
18. Saricaoglu M, Yilmaz NH, Ozer FF, Hanoglu L. The correlation of non-motor symptoms and sleep on balance in Parkinson's disease patients with normal cognition and mild cognitive impairment. *Irish Journal of Medical Science (1971-)* 2021;1–8.
19. Strouwen C, Molenaar EALM, Keus SHJ, et al. Protocol for a randomized comparison of integrated versus consecutive dual task practice in Parkinson's disease: the DUALITY trial. *BMC Neurol* 2014;14(1):1–12.
20. Roheger M, Kalbe E, Liepelt-Scarfone I. Progression of cognitive decline in Parkinson's disease. *J Parkinsons Dis* 2018;8(2):183–193.
21. Wallace ER, Segerstrom SC, Horne CG van, Schmitt FA, Koehl LM. Meta-analysis of cognition in Parkinson's disease mild cognitive impairment and dementia progression. *Neuropsychol Rev* 2021;1–12.
22. Segev-Jacubovski O, Herman T, Yogev-Seligmann G, Mirelman A, Giladi N, Hausdorff JM. The interplay between gait, falls and cognition: can cognitive therapy reduce fall risk? *Expert Rev Neurother* 2011;11(7):1057–1075.
23. Wood BH, Bilclough JA, Bowron A, Walker R. Incidence and prediction of falls in Parkinson's disease: a prospective multidisciplinary study. *J Neurol Neurosurg Psychiatry* 2002;72(6):721–725.
24. Allen NE, Sherrington C, Paul SS, Canning CG. Balance and falls in Parkinson's disease: a meta-analysis of the effect of exercise and motor training. *Movement disorders* 2011;26(9):1605–1615.
25. Wood BH, Bilclough JA, Bowron A, Walker R. Incidence and prediction of falls in Parkinson's disease: a prospective multidisciplinary study. *J Neurol Neurosurg Psychiatry* 2002;72(6):721–725.
26. Beck EN, Intzandt BN, Almeida QJ. Can dual task walking improve in Parkinson's disease after external focus of attention exercise? A single blind randomized controlled trial. *Neurorehabil Neural Repair* 2018;32(1):18–33.
27. Brauer SG, Morris ME. Can people with Parkinson's disease improve dual tasking when walking? *Gait Posture* 2010;31(2):229–233.
28. Brauer SG, Woollacott MH, Lamont R, et al. Single and dual task gait training in people with Parkinson's disease: a protocol for a randomised controlled trial. *BMC Neurol* 2011;11(1):1–6.
29. Dirnberger G, Jahanshahi M. Executive dysfunction in P arkinson's disease: A review. *J Neuropsychol* 2013;7(2):193–224.
30. Plummer P, Eskes G. Measuring treatment effects on dual-task performance: a framework for research and clinical practice. *Front Hum Neurosci* 2015;9:225.
31. Leone C, Feys P, Moumdjian L, D'Amico E, Zappia M, Patti F. Cognitive-motor dual-task interference: a systematic review of neural correlates. *Neurosci Biobehav Rev* 2017;75:348–360.
32. Fernandes Â, Coelho T, Vitória A, et al. Standing balance in individuals with Parkinson's disease during single and

dual-task conditions. *Gait Posture* 2015;42(3):323–328.
33. Bloem BR, Valkenburg V V, Slabbekoorn M, Dijk JG van. The multiple tasks test. Strategies in Parkinson's disease. *Exp Brain Res* 2001;137:478–486.
34. Bond JM, Morris M. Goal-directed secondary motor tasks: their effects on gait in subjects with Parkinson disease. *Arch Phys Med Rehabil* 2000;81(1):110–116.
35. O'Shea S, Morris ME, Iansek R. Dual task interference during gait in people with Parkinson disease: effects of motor versus cognitive secondary tasks. *Phys Ther* 2002;82(9):888–897.
36. Rochester L, Hetherington V, Jones D, et al. Attending to the task: interference effects of functional tasks on walking in Parkinson's disease and the roles of cognition, depression, fatigue, and balance. *Arch Phys Med Rehabil* 2004;85(10):1578–1585.
37. Salazar RD, Ren X, Ellis TD, et al. Dual tasking in Parkinson's disease: cognitive consequences while walking. *Neuropsychology* 2017;31(6):613.
38. Hsiu-Chen C, Chiung-Chu C, Jiunn-Woei L, et al. The effects of dual-task in patients with Parkinson's disease performing cognitive-motor paradigms. *Journal of Clinical Neuroscience* 2020;72:72–78.
39. Johansson H, Ekman U, Rennie L, Peterson DS, Leavy B, Franzén E. Dual-task effects during a motor-cognitive task in Parkinson's disease: patterns of prioritization and the influence of cognitive status. *Neurorehabil Neural Repair* 2021;35(4):356–366.
40. Broeder S, Nackaerts E, Nieuwboer A, Smits-Engelsman BCM, Swinnen SP, Heremans E. The effects of dual tasking on handwriting in patients with Parkinson's disease. *Neuroscience* 2014;263:193–202.
41. Criminger C, Swank C. Impact of dual-tasking on mobility tasks in Parkinson's disease as described through 2D kinematic analysis. *Aging Clin Exp Res* 2020;32:835–840.
42. Silva-Batista C, Corcos DM, Kanegusuku H, et al. Balance and fear of falling in subjects with Parkinson's disease is improved after exercises with motor complexity. *Gait Posture* 2018;61:90–97.
43. Falck RS, Davis JC, Best JR, Crockett RA, Liu-Ambrose T. Impact of exercise training on physical and cognitive function among older adults: a systematic review and meta-analysis. *Neurobiol Aging* 2019;79:119–130.
44. Herold F, Hamacher D, Schega L, Müller NG. Thinking while moving or moving while thinking–concepts of motor-cognitive training for cognitive performance enhancement. *Front Aging Neurosci* 2018;10:228.
45. Yang Y-R, Cheng S-J, Lee Y-J, Liu Y-C, Wang R-Y. Cognitive and motor dual task gait training exerted specific training effects on dual task gait performance in individuals with Parkinson's disease: A randomized controlled pilot study. *PLoS One* 2019;14(6):e0218180.
46. Canning CG. The effect of directing attention during walking under dual-task conditions in Parkinson's disease. *Parkinsonism Relat Disord* 2005;11(2):95–99.
47. Radder DLM, Sturkenboom IH, Nimwegen M van, Keus SH, Bloem BR, Vries NM de. Physical therapy and occupational therapy in Parkinson's disease. *International Journal of Neuroscience* 2017;127(10):930–943.
48. Basch E, Spertus J, Dudley RA, et al. Methods for developing patient-reported outcome-based performance measures (PRO-PMs). *Value in health* 2015;18(4):493–504.
49. Martinez-Martin P, Rodriguez-Blazquez C, Frades-Payo B. Specific patient-reported outcome measures for Parkinson's disease: analysis and applications. *Expert Rev Pharmacoecon Outcomes Res* 2008;8(4):401–418.
50. Leavy B, Löfgren N, Nilsson M, Franzén E. Patient-reported and performance-based measures of walking in mild–moderate Parkinson's disease. *Brain Behav* 2018;8(9):e01081.
51. McGee RG. How to include patient-reported outcome measures in clinical trials. *Curr Osteoporos Rep* 2020;18:480–485.
52. Chiarelli AM, Zappasodi F, Pompeo F Di, Merla A. Simultaneous functional near-infrared spectroscopy and electroencephalography for monitoring of human brain activity and oxygenation: a review. *Neurophotonics* 2017;4(4):41411.
53. Kahya M, Moon S, Ranchet M, et al. Brain activity during dual task gait and balance in aging and age-related neurodegenerative conditions: a systematic review. *Exp Gerontol* 2019;128:110756.
54. Cao J, Garro EM, Zhao Y. EEG/FNIRS based workload classification using functional brain connectivity and machine learning. *Sensors* 2022;22(19):7623.
55. Maidan I, Nieuwhof F, Bernad-Elazari H, et al. The role of the frontal lobe in complex walking among patients with Parkinson's disease and healthy older adults: an fNIRS study. *Neurorehabil Neural Repair* 2016;30(10):963–971.
56. Herold F, Wiegel P, Scholkmann F, Müller NG. Applications of functional near-infrared spectroscopy (fNIRS) neuroimaging in exercise–cognition science: a systematic, methodology-focused review. *J Clin Med* 2018;7(12):466.
57. Obrig H. NIRS in clinical neurology—a 'promising'tool? *Neuroimage* 2014;85:535–546.
58. Bonilauri A, Sangiuliano Intra F, Pugnetti L, Baselli G, Baglio F. A systematic review of cerebral functional near-infrared spectroscopy in chronic neurological diseases—actual applications and future perspectives. *Diagnostics* 2020;10(8):581.
59. Nieuwhof F, Reelick MF, Maidan I, et al. Measuring prefrontal cortical activity during dual task walking in patients with Parkinson's disease: feasibility of using a new portable fNIRS device. *Pilot Feasibility Stud* 2016;2(1):1–11.
60. Dorsey ER, Elbaz A, Nichols E, et al. Global, regional, and national burden of Parkinson's disease, 1990–2016: a systematic analysis for the Global Burden of Disease Study 2016. *Lancet Neurol* 2018;17(11):939–953.
61. Leiva AM, Martínez-Sanguinetti MA, Troncoso-Pantoja C, Nazar G, Petermann-Rocha F, Celis-Morales C. Chile

lidera el ranking latinoamericano de prevalencia de enfermedad de Parkinson. *Rev Med Chil* 2019;147(4):535–536.
62. Goodarzi Z, Mrklas KJ, Roberts DJ, Jette N, Pringsheim T, Holroyd-Leduc J. Detecting depression in Parkinson disease: a systematic review and meta-analysis. *Neurology* 2016;87(4):426–437.
63. Campos-Romero S, Lucchini Raies C, Rodríguez C, Muñoz P, Márquez-Doren F, Bustamante-Troncoso C. Adaptación cultural y validación del instrumento de Autoeficacia para el manejo de enfermedades crónicas en población chilena. *Rev Med Chil* 2023;151(12):1596–1603.
64. Clavijo M, Yévenes F, Gallardo I, Contreras AM, Santos C. Escala de autoeficacia general: reevaluación de su evidencia de confiabilidad y validez en Chile. *Rev Med Chil* 2020;148(10):1452–1460.
65. Bustamante-Contreras C, Ojeda-Gallardo Y, Rueda-Sanhueza C, Rossel PO, Martínez-Carrasco C. Spanish version of the mini-BESTest: a translation, transcultural adaptation and validation study in patients with Parkinson's disease. *International Journal of Rehabilitation Research* 2020;43(2):129–134.
66. Janssen S, Heijs JJA, Meijs W Van Der, et al. Validation of the Auditory Stroop Task to increase cognitive load in walking tasks in healthy elderly and persons with Parkinson's disease. *PLoS One* 2019;14(8):e0220735.
67. Strouwen C, Molenaar EALM, Keus SHJ, Münks L, Bloem BR, Nieuwboer A. Test-retest reliability of dual-task outcome measures in people with Parkinson disease. *Phys Ther* 2016;96(8):1276–1286.
68. Gaudet P. Measuring the impact of Parkinson's disease: an occupational therapy perspective. *Canadian Journal of Occupational Therapy* 2002;69(2):104–113.
69. Kobayashi E, Himuro N, Mitani Y, Tsunashima T, Nomura K, Chiba S. Feasibility and informativeness of the Canadian occupational performance measure for identifying priorities in patients with Parkinson's disease. *Physiother Theory Pract* 2023;39(3):607–614.
70. Martínez-Martín P, Payo BF, Disorders GC for S of M. Quality of life in Parkinson's disease: validation study of the PDQ-39 Spanish version. *J Neurol* 1998;245:S34–S38.
